# Supplementary material for: Deletion of Smooth Muscle O-GlcNAc Transferase Prevents Development of Atherosclerosis in Western Diet-Fed Hyperglycemic ApoE-/- Mice In Vivo
Source: Int J Mol Sci. 2023 Apr 26;24(9):7899. doi: 10.3390/ijms24097899 (PMC10178779; doi:10.3390/ijms24097899)
Supplement: Supplementary file 1 [file ijms-24-07899-s001.zip › ijms-2344696-supplementary.pdf]

A) The primer sequences used for genotyping and expected size of PCR products are as follows:

1. **OGT**: Mutant: 487 bp; WT: 338 bp
   F- CAT CTC TCC AGC CCC ACA AAC TG
   R- GAC GAA GCA GGA GGG GAG AGC AC

2. **ApoE**: Mutant: 245 bp; WT: 155 bp
   Common GCC TAG CCG AGG GAG AGC CG
   Wild type Reverse TGT GAC TTG GGA GCT CTG CAG C

3. ***Myh11-CreER***^{T2}: Transgene: 287 bp; Internal positive control: 180 bp
   Tran F- TGA CCC CAT CTC TTC ACT CC
   Tran R- AGT CCC TCA CAT CCT CAG GTT
   Positive control F- CAG CCA ACT TTA CGC CTA GC
   Positive control R- TCT CAA GAT GGA CCT AAT ACG G

B) PCR Cycling conditions

1. For Cre^{tg} and OGT^{F}:

Temperature	Time	Repetitions
94°C	5 min	
94°C	20s	
65-55°C	15s	10X (lower 1C each cycle)
68°C	10s	
94°C	15s	
60°C	15s	30X
72°C	10s	
72°C	2 min	
15°C	hold	

2. For ApoE:

Temperature	Time	Repetition
94°C	4 min	
94°C	30s	
69°C	1 min	35X
72°C	1 min	
72°C	2 min	
15°C	hold	

Supplementary Figure S1. Primer sequences and PCR conditions for mice genotyping. A) Shown are the primer sequences used for OGT, ApoE and Myh11-Cre^{tg} genotyping, B) PCR cycling conditions used for mice genotyping.

A. Primer sequences utilized for qPCR reactions:

	Gene	Forward	Reverse
1	*Acta2*	5'- CATCTTTCATTGGGATGGAG- 3'	5'- TTAGCATAGAGATCCTTCCTG- 3'
3	*Myocd*	5'- GATGGGCTCTCTCCAGATCAG- 3'	5'- GGCTGCATCATTCTTGTCACTT- 3'
4	*Myh11*	5' TATCTTCTGGAAAAGTCTAGGG- 3'	5'- AAAGCAGGTCACTTTTCATC- 3'
6	*36b4*	5'- AGATTCGGGATATGCTGTTGG – 3'	5'- TCGGGTCCTAGACCAGTGTTC – 3'
7	*18s*	5'- TTGACGGAAGGGCACCACCAG- 3'	5'- GCACCACCACCCACGGAATCG- 3'
8	*OGT*	5'- GACGCAACCAAACTTTGCAGT-3'	5'-TCAAGGGTGACAGCCTTTTCA- 3'
9	*Cnn1*	5'- TGCTGAAGTAAAGAACAAGC- 3'	5'- CATTGACCTTCTTCACAGAAC- 3'
10	*Il6*	5'- TAGTCCTTCCTACCCCAATTTCC- 3'	5'- TTGGTCCTTAGCCACTCCTTC- 3'
11	*Il1β*	5'- TGTAATGAAAGACGGCACACC- 3'	5'- TCTTCTTTGGGTATTGCTTGG- 3'

B. qPCR cycling conditions

Thermal Cycler Profile			
Stage	Repetitions	Temperature	Time (mins)
1	1	50.0 °C	2:00
2	1	95.0 °C	10:00
3	40	95.0 °C	0:15
		60.0 °C	1:00
4 (Dissociation)	1	95.0 °C	0:15
		60.0 °C	0:20
		95.0 °C	0:15
		60.0 °C	0:15

Supplementary Figure S2. Real-time PCR conditions. A) qPCR primer sequences and B) qPCR cycling conditions.

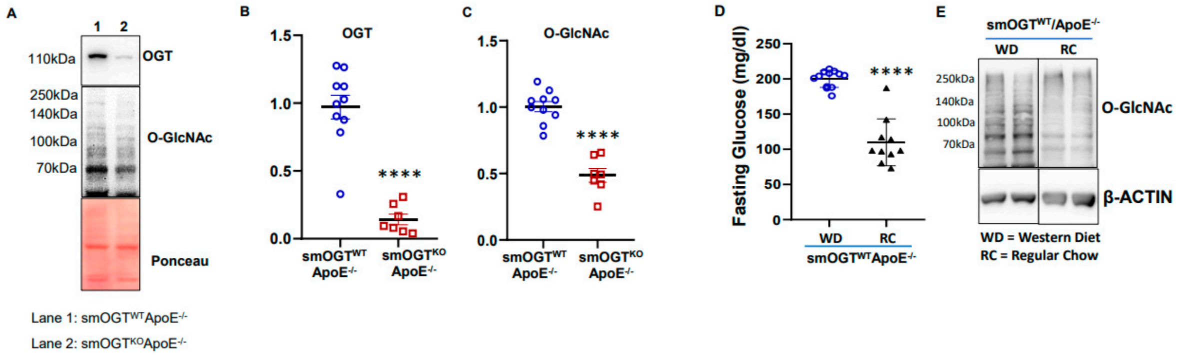

Supplementary Figure S3. (A-C) Validation of SMC-specific OGT deletion in ApoE$^{-/-}$ mice. Aortic lysates derived from smOGT^{WT}ApoE$^{-/-}$ and smOGT^{KO}ApoE$^{-/-}$ mice were subjected to immunoblotting. Shown are representative immunoblots and corresponding summary graphs depicting fold-change in protein expression normalized to Ponceau S for total protein staining. D-E) Western diet feeding increases fasting blood glucose levels accompanied with increased O-GlcNAc protein expression in the aortic vessels of smOGT^{WT}ApoE$^{-/-}$ mice. n=7-11 mice per group, $^{****}p<0.0001$.

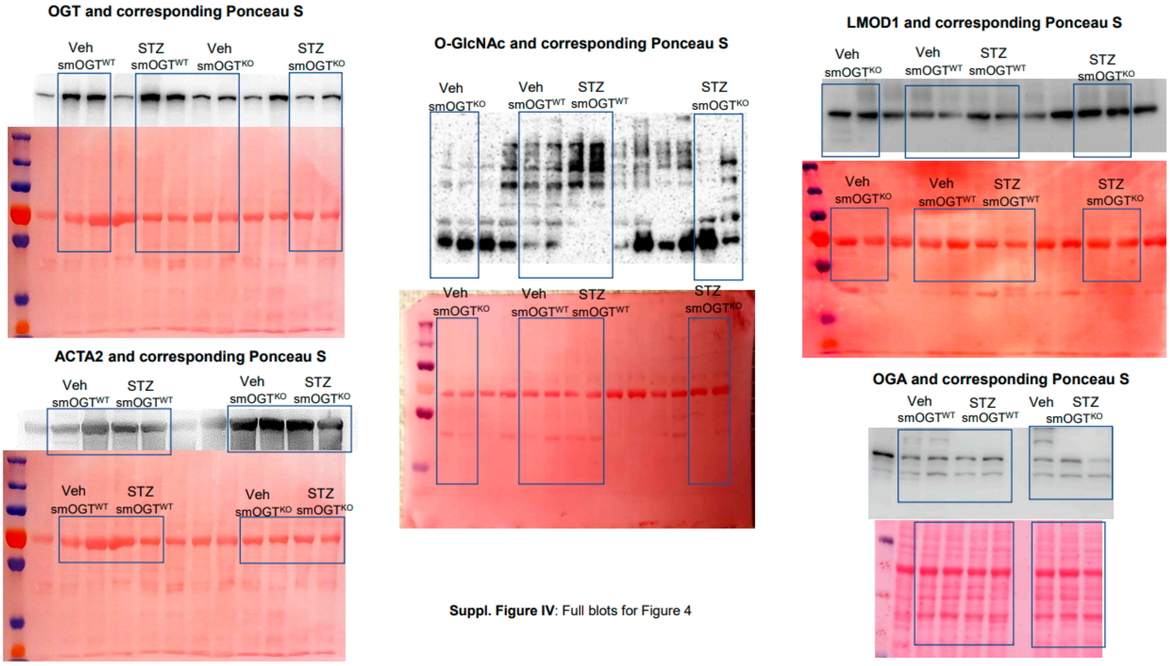

Supplementary Figure S4. Full blots for Figure 4 images. Blue box outlines the bands used in the corresponding images.

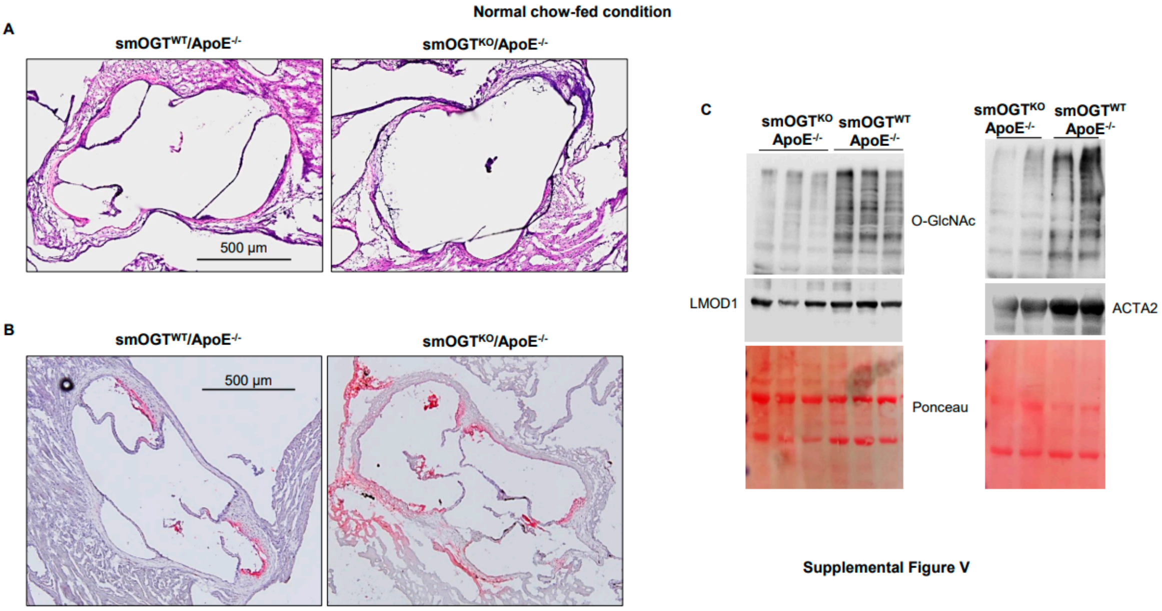

Supplementary Figure S5. Plaque area, lipid burden and SM contractile marker expression in normal chow-fed smOGT^{WT}ApoE$^{-/-}$ and smOGT^{KO}ApoE$^{-/-}$ mice. Shown are representative A) hematoxylin & eosin (H&E)- and B) Oil red O-stained images of aortic root sections derived from 14-wks old normal chow-fed smOGT^{WT}ApoE$^{-/-}$ and smOGT^{KO}ApoE$^{-/-}$ mice. Representative immunoblots of aortic lysates from normal chow-fed smOGT^{WT}ApoE$^{-/-}$ and smOGT^{KO}ApoE$^{-/-}$ mice depicting O-GlcNAc, LMOD1 and ACTA2 expression are shown in panel C); Ponceau S shows total protein staining.

Supplementary material: The following Supplemental data are included in this manuscript: Supplementary Figures S1-S5.
